# Bullying and cyberbullying victimization and associated mental health and behavioral risks among adolescents in Ecuador

**DOI:** 10.3389/fsoc.2026.1803370

**Published:** 2026-04-28

**Authors:** Martha Fors, Paloma Gonzalez, Ariel Torres, Fresia Massuht

**Affiliations:** 1 One Health Research Group, Facultad de Ciencias de la Salud, Universidad de las Américas, Quito, Ecuador; 2 Biblioteca, Universidad de las Américas, Quito, Ecuador; 3 Facultad de Ciencias de la Salud y Bienestar Humano, Universidad Tecnológica Indoamérica, Quito, Ecuador; 4 IESS Seguro de Salud Centro aa. Sur Valdivia, Guayaquil, Ecuador

**Keywords:** adolescents, bullying, cyberbullying, ecuador, mental health, suicide risk

## Abstract

**Introduction:**

Bullying remains a major public health concern among adolescents and is associated with adverse mental health and behavioral outcomes. Evidence from low- and middle-income countries, including Ecuador, remains limited.

**Methods:**

We conducted a cross-sectional study using data from the Ecuadorian version of the Youth Risk Behavior Surveillance System (YRBSS). Associations between school bullying, cyberbullying, and emotional distress (sadness), suicidal behaviors (ideation, planning, attempts), and risk behaviors (alcohol use, marijuana use, and school fights) were assessed using chi-square tests and logistic regression. Odds ratios (OR) and relative risks (RR) with 95% confidence intervals (CI) were calculated.

**Results:**

Among 300 adolescents, 10.0.3% reported school bullying and 16.0% reported cyberbullying. Bullying victimization was associated with higher odds of sadness (OR 3.33, 95% CI 1.50–7.35), suicidal ideation (OR 431, 1.99–9.0.32), suicidal planning (OR 5.45, 2.51–1183), and suicide attempts (OR 5.43, 2.39–12.36). Cyberbullying was strongly associated with alcohol consumption (RR 5.33, 95% CI 2.47–11.46) and marijuana use (RR 7.20, 1.86–27.92).

**Discussion:**

Bullying and cyberbullying are strongly associated with emotional distress, suicidal behaviors, and substance use among adolescents in Ecuador. These findings underscore the urgent need for school-based prevention strategies and integrated public health interventions targeting adolescent mental health.

## 1 Background

Bullying is defined by the World Health Organization as repeated physical, psychological, or sexual intimidation that harms a victim's emotional and social wellbeing (Castro-Robles et al., 2020). It is a major public health and educational concern worldwide, associated with depression, anxiety, substance use, school disengagement, and suicidal behavior (Romero, 2018; Castañeda-Vázquez et al., 2020).

School bullying and cyberbullying are forms of peer victimization that occur in both physical and digital environments. These experiences represent important psychosocial stressors during adolescence, a developmental stage characterized by heightened emotional sensitivity and strong peer influence. Evidence suggests that repeated exposure to peer victimization is associated with a range of adverse outcomes, including emotional distress, depressive symptoms, substance use, and suicidal behaviors. Understanding bullying within the broader framework of peer victimization provides a conceptual basis for examining how hostile interactions in school and online contexts may affect adolescent mental health and risk behaviors.

Globally, the prevalence of bullying and cyberbullying varies substantially, ranging from approximately 25% in the Caribbean to over 48% in Sub-Saharan Africa (UNESCO, 2019). In the United States, 14.9% of adolescents report experiencing cyberbullying and 13.6% report a history of serious suicide attempts, underscoring ongoing concerns regarding adolescent mental health (Schonfeld et al., 2023). Prior research consistently shows that involvement in bullying is associated with increased anxiety, depression, psychosocial difficulties, and self-harming behaviors.

School bullying and cyberbullying represent peer victimization occurring in physical and digital environments, acting as psychosocial stressors during adolescence when peer influence peaks. Despite this international evidence, research examining bullying and cyberbullying in Ecuador remains limited. National reports indicate that bullying negatively affects school performance and adolescent wellbeing (Rojas et al., 2023), yet few studies have explored its association with emotional distress and behavioral risks. Ecuadorian adolescents face persistent challenges related to mental health service access and school-based support, alongside rising concerns regarding substance use, depressive symptoms, and suicidal behaviors.

This study aimed to examine the association between school bullying and cyberbullying victimization and adverse emotional and behavioral outcomes, including sadness, suicidal behaviors, and substance use, among Ecuadorian adolescents using nationally validated survey data.

## 2 Methods

### 2.1 Study design and participants

A cross-sectional analytical study was conducted using data from the Ecuadorian adaptation of the Youth Risk Behavior Surveillance System (EYRBSS) to examine the association between bullying victimization (school bullying and cyberbullying) and emotional distress, suicidal behaviors, and risk behaviors among adolescents. Participants were recruited from secondary schools using a convenience sampling approach. Schools that agreed to participate were contacted by the research team, and students present on the day of data collection were invited to complete the anonymous survey. Although this sampling strategy does not allow national representativeness, it is commonly used in school-based epidemiological studies aimed at exploring associations between behavioral and mental health variables. Participation was voluntary and anonymous. Data was collected directly by the authors through a structured, self-administered survey distributed in participating schools. Given the exploratory nature of the study, no formal *a priori* power analysis was conducted. However, the sample size was considered sufficient to detect moderate associations between bullying victimization and behavioral outcomes using chi-square and regression analyses.

### 2.2 Instrument

The Spanish version of the Youth Risk Behavior Surveillance System (YRBSS) was used, previously validated in Ecuador through a descriptive, cross-sectional study of adolescents. This validation confirmed the instrument's suitability for assessing risk behaviors, including bullying victimization, emotional distress (sadness), suicidal behaviors (ideation/planning), and substance use (alcohol/marijuana), demonstrating adequate psychometric properties for the national context.

### 2.3 Measures

Bullying victimization (dependent variable) was assessed through a self-reported dichotomous item (yes/no). Independent variables included indicators of emotional distress and risk behaviors during the previous 12 months: persistent sadness (lasting ≥2 weeks), suicidal ideation, suicidal planning, suicide attempts, lifetime alcohol consumption, and lifetime marijuana use. Age (continuous) and sex (binary) were considered as potential confounders.

#### 2.3.1 Variables

Bullying was assessed with: “During the past 12 months, have you ever been bullied on school property?”

Cyberbullying was assessed with: “During the past 12 months, have you ever been electronically bullied?”

Sadness was defined by the YRBSS item: “During the past 12 months, did you ever feel so sad or hopeless almost every day for two weeks or more in a row that you stopped doing some usual activities?”

A total of 415 respondents were obtained, and from the secondary database, the sample selection was carried out:

A total of 415 questionnaires were collected directly by the research team from consenting secondary schools using convenience sampling. For the present analysis, complete case analysis was applied, retaining only participants with non-missing data on all key variables: school bullying, cyberbullying, sadness, suicidal ideation/planning/attempts, alcohol use, and marijuana use (*n* = 9 exclusions per variable minimum). Total exclusions: 115 questionnaires (27.7%) due to incomplete responses on ≥1 key variable. Final analytic sample: 300 adolescents with complete data across all exposures/outcomes. Missingness patterns: No differential missingness by bullying status *(*χ^2^ = 1.24, *p* = 0.27) or primary outcomes (all *p* > 0.10), minimizing selection bias.

The variables under analysis were gender, age, fights at school, alcohol consumption, Marijuana use, sadness, suicidal thoughts, suicidal planning, and suicidal attempts according to school bullying and cyberbullying.

Fighting at school was explored with “During the past 12 months, how many times were you in a physical fight on school property? While alcohol consumption was evaluated by “During the past 30 days, on how many days did you have at least one drink of alcohol? Marijuana use was assessed by asking: “During your life, how many times have you used marijuana?”

Sadness, suicide thoughts, and suicide planning were assessed with the questions: “During the past 12 months, did you ever feel so sad or hopeless almost every day for two weeks or more in a row that you stopped doing some usual activities?”, “During the past 12 months, did you ever seriously consider attempting suicide?”, and “During the past 12 months, did you make a plan about how you would attempt suicide?” A suicide attempt was assessed by asking the question: “During the past 12 months, how many times did you actually attempt suicide?”

The main independent variable was bullying at school was assessed with the question: “During the past 12 months, have you ever been bullied on school property?” Cyberbullying was assessed with the item: “During the past 12 months, have you ever been electronically bullied? (messages, photos, videos in WhatsApp, Facebook, Instagram, twitter or other social media)

### 2.4 Statistical analysis

Descriptive statistics were used to summarize the characteristics of the study population. Categorical variables were expressed as frequencies and percentages. Associations between school bullying and cyberbullying were examined using Pearson's chi-square test.

Bivariate analyses were conducted to assess associations between bullying victimization (school bullying and cyberbullying) and behavioral and emotional factors, including alcohol consumption, marijuana use, involvement in school fights, sadness, suicidal thoughts, suicidal plans, and suicide attempts. For these analyses, chi-square tests were used to evaluate statistical significance, and measures of association were estimated using relative risks (RR) or odds ratios (OR), with corresponding 95% confidence intervals.

Gender differences in bullying and cyberbullying victimization were examined by estimating relative risks with 95% confidence intervals, comparing female and male adolescents. All tests were two-sided, and a *p*-value < 0.05 was considered statistically significant. Multivariable logistic regression models were conducted to evaluate whether the associations between bullying victimization and emotional and behavioral outcomes remained significant after adjusting for potential confounders. Age and sex were included as covariates in the adjusted models. Adjusted odds ratios (aOR) with 95% confidence intervals were calculated. Effect estimates from contingency table analyses were reported as relative risks (RR) or odds ratios (OR) depending on the distribution of the variables. Multivariable analyses were conducted using logistic regression models; therefore, adjusted odds ratios (aOR) with 95% confidence intervals were reported for these models.

Statistical analyses were performed using standard statistical software SPSS software version 27.

### 2.5 Ethics aspects

The study protocol was approved by the institutional Ethics Committee (name and approval reference removed for double-blind peer review per journal policy). Adolescents provided informed assent, and parents/legal guardians gave written informed consent prior to survey participation. All participants were assured complete anonymity and confidentiality, with voluntary participation and unrestricted right to withdraw without consequence. Procedures adhered to the Declaration of Helsinki ethical standards for human subjects research.

## 3 Results

A total of 300 adolescents were included in the analysis. Overall, 10.3% (*n* = 31) reported having experienced school bullying, while 16.0% (*n* = 48) reported cyberbullying. A significant association was observed between school bullying and cyberbullying (*p* = 0.019). Adolescents who experienced school bullying were more likely to also report cyberbullying compared with those who were not bullied at school (Table 1), indicating overlap between in-person and online victimization. Adolescents who reported being bullied at school had higher odds of cyberbullying compared with those who did not report school bullying (OR 3.0.45; 95% CI 1.0.55–7.0.69; χ^2^ = 9.0.77; *p* = 0.0.002).

**Table 1:** Association between school bullying and cyberbullying among adolescents (*n* = 300).

Type of bullying	Cyberbullying		Total	*p* value^*^
School bullying	Yes *n* (%)	No *n* (%)	*n* (%)	
Yes	10 (3.3%)	21 (7.0%)	31 (10.3%)	0.019
No	38 (12.7%)	231 (77.0%)	269 (89.7%)	
Total	48 (16.0%)	252 (84.0%)	300(100%)	

^*^Chi square test.

### 3.1 Gender differences

**Figure 1:**
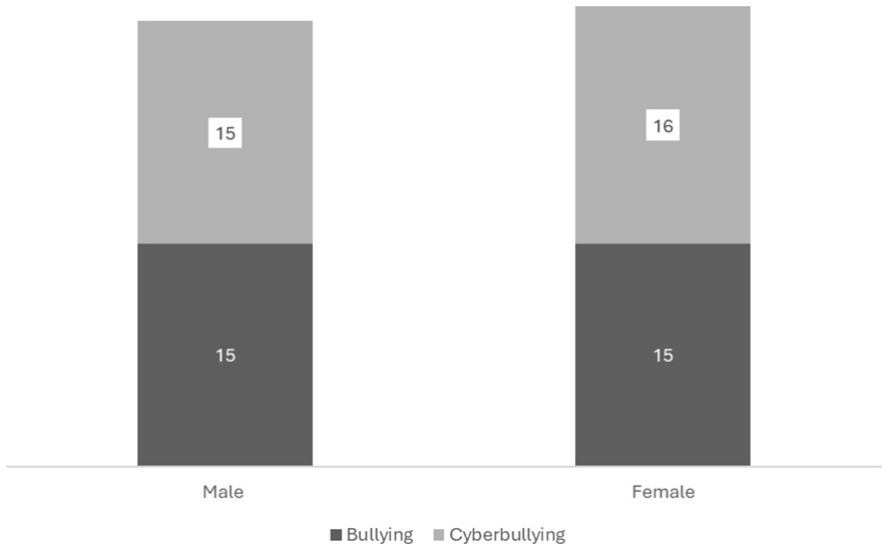
Gender differences in bullying victimization. Gender differences in school bullying (M: 15/155, F: 16/145; RR 0.60, *p* = 0.134) and cyberbullying (M: 15/155, F: 16/145; RR 0.70, *p* = 0.110) prevalence (*n* = 300). No significant sex differences.

No statistically significant differences were observed between sexes for either form of bullying victimization. For school bullying, the relative risk for females compared to males was 0.60 (95% CI: 0.32–1.12; *p* = 0.134), while for cyberbullying the relative risk was 0.70 (95% CI: 0.33–1.47; *p* = 0.110). These findings indicate similar exposure to bullying and cyberbullying among male and female adolescents (Figure 1).

### 3.2 School bullying and associated risk factors

School bullying was significantly associated with several behavioral and emotional outcomes (Table 2). Adolescents who reported alcohol consumption were more likely to experience school bullying compared to non-drinkers (13.8% vs. 6.8%; *p* = 0.042; OR = 2.21, 95% CI: 1.01–4.87). Involvement in school fights was also more frequent among bullied students (23.4% vs. 7.9%; *p* = 0.005; OR = 3.44, 95% CI: 1.52–7.77).

**Table 2:** Associations between school bullying and emotional and behavioral risk factors among adolescents (*n* = 300).

Bullying					
Variable	Yes *n* (%)	No *n* (%)	Total (*n*)	*p*-value^*^	OR (95% CI)
Alcohol consumption	21 (13.8%)	131 (86.2%)	152	0.042	2.21 (1.01–4.87)
Marijuana use	2 (22.2%)	7 (77.8%)	9	0.290	2.58 (0.51–13.01)
Fighting at school	11 (23.4%)	36 (76.6%)	47	0.005	3.44 (1.52–7.77)
Sadness	21 (16.8%)	104 (83.2%)	125	0.002	3.33 (1.50–7.35)
Suicidal thoughts	15 (23.8%)	48 (76.2%)	63	<0.001	4.31 (1.99–9.32)
Suicidal plans	16 (26.7%)	44 (73.3%)	60	<0.001	5.45 (2.51–11.83)
Suicide attempts	12 (30.0%)	28 (70.0%)	40	<0.001	5.43 (2.39–12.36)

^*^Chi-square test. RR, relative risk; CI, confidence interval.

Emotional distress was significantly higher among adolescents exposed to bullying. Sadness was reported by 16.8% of bullied students compared to 5.7% of non-bullied peers (*p* = 0.002; OR = 3.33, 95% CI: 1.50–7.35). Strong associations were observed between school bullying and suicidal behaviors. Adolescents who experienced bullying were more likely to report suicidal thoughts (23.8% vs. 6.8%; *p* < 0.001; OR = 4.31), suicidal plans (26.7% vs. 6.3%; *p* < 0.001; OR = 5.45), and suicide attempts (30.0% vs. 7.3%; *p* < 0.001; OR = 5.43). Marijuana use was not significantly associated with school bullying.

Additional bivariate associations between school bullying and behavioral outcomes are presented in Table 3, showing that school bullying was associated with a two- to five-fold increased risk of emotional distress and suicidal behaviors. The strongest associations were observed for suicidal plans (RR = 5.45) and suicide attempts (RR = 5.43). No statistically significant association was observed between school bullying and marijuana use.

**Table 3:** Bivariate associations between school bullying and emotional and behavioral outcomes among adolescents (*n* = 300).

School bullying					
Variable	Yes *n* (%)	No *n* (%)	Total (*n*)	*p*-value^*^	OR (95% CI)
Alcohol consumption					
Yes	21 (13.8%)	131 (86.2%)	152	0.042	2.21 (1.01–4.87)
No	10 (6.8%)	138 (93.2%)	148		
Marijuana use					
Yes	2 (22.2%)	7 (77.8%)	9	0.29	2.58 (0.51–13.01)
No	29 (10.0%)	262 (90.0%)	291		
Fighting at school					
Yes	11 (23.4%)	36 (76.6%)	47	0.005	3.44 (1.52–7.77)
No	20 (7.9%)	232 (92.1%)	252		
Sadness					
Yes	21 (16.8%)	104 (83.2%)	125	0.002	3.33 (1.50–7.35)
No	10 (5.7%)	165 (94.3%)	175		
Suicidal thoughts					
Yes	15 (23.8%)	48 (76.2%)	63	<0.001	4.31 (1.99–9.32)
No	16 (6.8%)	221 (93.2%)	237		
Suicidal plans					
Yes	16 (26.7%)	44 (73.3%)	60	<0.001	5.45 (2.51–11.83)
No	15 (6.3%)	225 (93.8%)	240		
Suicidal attempts					
Yes	12 (30.0%)	28 (70.0%)	40	<0.001	5.43 (2.39–12.36)
No	19 (7.3%)	241 (92.7%)	260		

* Chi-square test. RR, relative risk; CI, confidence interval.

### 3.3 Cyberbullying and associated risk factors

Cyberbullying showed strong associations with multiple behavioral and emotional risk factors (Table 4). Adolescents who reported alcohol consumption had a significantly higher likelihood of experiencing cyberbullying compared with non-drinkers (25.7% vs. 6.1%; *p* < 0.001; RR = 5.33, 95% CI: 2.47–11.46). Marijuana use was also strongly associated with cyberbullying, with users being over seven times more likely to report cyberbullying victimization (RR = 7.20, 95% CI: 1.86–27.92; *p* = 0.001).

**Table 4:** Associations between cyberbullying victimization and emotional and behavioral outcomes among adolescents (*n* = 300).

Cyberbullying					
Variable	Yes *n* (%)	No *n* (%)	Total (*n*)	*p*-value	RR (95% CI)
Alcohol consumption					
Yes	39 (25.7%)	113 (74.3%)	152	<0.001	5.33 (2.47–11.46)
No	9 (6.1%)	139 (93.9%)	148		
Marijuana use					
Yes	5 (55.6%)	4 (44.4%)	9	0.001	7.20 (1.86–27.92)
No	43 (14.8%)	248 (85.2%)	291		
Fighting at school					
Yes	15 (31.3%)	33 (68.8%)	48	0.003	3.01 (1.48–6.14)
No	28 (11.1%)	224 (88.9%)	252		
Sadness					
Yes	36 (28.8%)	89 (71.2%)	125	<0.001	5.49 (2.72–11.09)
No	12 (6.9%)	163 (93.1%)	175		
Suicidal thoughts					
Yes	20 (31.7%)	43 (68.3%)	63	<0.001	3.47 (1.79–6.72)
No	28 (11.8%)	209 (88.2%)	237		
Suicidal plans					
Yes	20 (33.3%)	40 (66.7%)	60	<0.001	3.78 (1.94–7.36)
No	28 (11.7%)	212 (88.3%)	240		
Suicidal attempts					
Yes	14 (35.0%)	26 (65.0%)	40	0.001	3.57 (1.70–7.52)
No	34 (13.1%)	226 (86.9%)	260		

^*^Chi-square test. RR, relative risk; CI, confidence interval.

Participation in school fights was associated with an increased risk of cyberbullying (31.3% vs. 11.1%; *p* = 0.003; RR = 3.01, 95% CI: 1.48–6.14). Emotional distress demonstrated a similar pattern: sadness was reported by 28.8% of cyberbullied adolescents compared with 6.9% of non-cyberbullied peers (*p* < 0.001; RR = 5.49, 95% CI: 2.72–11.09).

Cyberbullying was also significantly associated with suicidal behaviors. Adolescents experiencing cyberbullying reported higher prevalence of suicidal thoughts (31.7% vs. 11.8%; *p* < 0.001; RR = 3.47), suicidal plans (33.3% vs. 11.7%; *p* < 0.001; RR = 3.78), and suicide attempts (35.0% vs. 13.1%; *p* = 0.001; RR = 3.57), compared with those who did not report cyberbullying.

### 3.4 School bullying and associated risk factors

To further examine whether these associations remained significant after accounting for demographic characteristics, multivariable logistic regression models adjusted for age and sex were conducted. The adjusted analyses showed that school bullying remained significantly associated with sadness (aOR 3.05, 95% CI 1.34–6.92), suicidal ideation (aOR 3.88, 95% CI 1.73–8.69), suicidal planning (aOR 4.92, 95% CI 2.18–11.09), and suicide attempts (aOR 4.61, 95% CI 1.95–10.87). Similarly, cyberbullying remained independently associated with sadness (aOR 4.78, 95% CI 2.24–10.18), suicidal ideation (aOR 3.12, 95% CI 1.58–6.17), suicidal planning (aOR 3.41, 95% CI 1.71–6.80), and suicide attempts (aOR 3.29, 95% CI 1.52–7.12). These results indicate that the associations observed in the bivariate analyses persisted after adjustment for age and sex (Table 5).

**Table 5:** Multivariable logistic regression models examining the association between bullying victimization and mental health outcomes among adolescents.

Outcome	Predictor	OR adjust	IC95%	*p*
Sadness	School bullying	3.05	1.34–6.92	0.007
Suicidal ideation	School bullying	3.88	1.73–8.69	<0.001
Suicidal planning	School bullying	4.92	2.18–11.09	<0.001
Suicide attempt	School bullying	4.61	1.95–10.87	<0.001
Sadness	Cyberbullying	4.78	2.24–10.18	<0.001
Suicidal ideation	Cyberbullying	3.12	1.58–6.17	0.001
Suicidal planning	Cyberbullying	3.41	1.71–6.80	<0.001
Suicide attempt	Cyberbullying	3.29	1.52–7.12	0.002

## 4 Discussion

This study identified robust associations between school bullying, cyberbullying, and multiple emotional and behavioral risk factors among Ecuadorian adolescents. The significant overlap between school bullying and cyberbullying supports the conceptualization of adolescent victimization as a cumulative phenomenon, consistent with the poly-victimization framework. Adolescents exposed to traditional bullying appeared more vulnerable to online victimization, reinforcing the need for prevention strategies that simultaneously address offline and digital contexts.

Secondary schools function as welfare institutions mediating peer victimization, yet face under-resourcing and uneven anti-bullying implementation. Urban schools' digital infrastructure elevates cyberbullying risk (smartphone ownership 85%), while rural schools confront overcrowding and mental health service gaps. These institutional realities shape victimization opportunities and coping resources unavailable in this dataset.”

The multivariable analyses confirmed that the associations observed in the bivariate models remained significant after adjusting for age and sex. School bullying and cyberbullying were independently associated with emotional distress and suicidal behaviors among adolescents. These findings strengthen the interpretation that bullying victimization represents an important risk factor for adverse mental health outcomes in this population.

In this sample, 10.3% of adolescents reported school bullying, a prevalence lower than that reported in several international studies (Schonfeld et al., 2023; John et al., 2018; Zaborskis et al., 2019; Nagata et al., 2023). In contrast, cyberbullying affected 16% of participants, exceeding prevalence estimates reported in recent studies (Chudal et al., 2022). These findings suggest that digital forms of victimization may be particularly salient in this population, possibly reflecting increased internet use and reduced supervision in online environments.

Substance use emerged as an important correlate of victimization. Alcohol consumption was significantly associated with both school bullying and cyberbullying, consistent with prior evidence suggesting that alcohol use increases vulnerability to victimization by lowering inhibition and increasing engagement in risky social contexts (Kowalski et al., 2014). Marijuana use was not associated with school bullying but showed a strong relationship with cyberbullying, in line with studies indicating that substance use may exacerbate emotional vulnerability and exposure to online risk behaviors (Azevedo da Silva and Martins, 2020; Sampasa-Kanyinga et al., 2022; Priesman et al., 2018).

Emotional distress was markedly higher among adolescents exposed to bullying. Sadness—a construct closely linked to pessimism and emotional dissatisfaction (Torres Salcedo et al., 2023; Laiza, 2023)—was significantly more common among victims of both school bullying and cyberbullying. These findings are consistent with evidence identifying bullying as a chronic stressor that disrupts emotional regulation and wellbeing.

Of particular concern were the strong associations between bullying and suicidal behaviors. Adolescents who experienced bullying showed substantially higher risks of suicidal ideation, planning, and attempts, with effect sizes exceeding those observed for sadness, substance use, or aggressive behaviors. Importantly, these associations remained statistically significant in the multivariable models adjusted for age and sex, suggesting that bullying victimization may independently contribute to increased suicide risk among adolescents. These findings echo international literature linking bullying to elevated suicide risk (Rodríguez Márquez et al., 2020). Similar associations have been reported in Colombia, where school bullying has been legally recognized as a serious offense due to its link with adolescent suicide (Valbuena Herrera, 2020; Palacio Durango and Espinosa Restrepo, 2022). Likewise, a study from the University of Santiago de Compostela reported a 2.36–3.06-fold increase in suicide risk among bullied adolescents (Azúa Fuentes et al., 2020), slightly lower than the relative risks observed in the present study.

The prominence of suicidal behaviors among bullied adolescents underscores the importance of viewing bullying not merely as a disciplinary or educational issue but as a critical mental health concern. While this study did not assess other adverse experiences such as family dysfunction or sexual violence, the findings raise important questions regarding cumulative exposure to multiple forms of abuse and their potential amplification of mental health risks.

Current intervention strategies for bullying and cyberbullying primarily focus on educational approaches involving schools and families. Technology-based prevention programs have also emerged, leveraging the same digital platforms used by adolescents. Evidence suggests that integrated interventions combining educational, psychosocial, and digital strategies may be more effective than isolated approaches (Tozzo et al., 2022). Addressing bullying effectively requires coordinated efforts among educators, mental health professionals, families, and digital specialists.

Overall, bullying and cyberbullying were strongly associated with emotional distress, suicidal behaviors, alcohol use, absenteeism, and involvement in fights. These interconnected outcomes highlight the multifaceted impact of victimization and emphasize the necessity of comprehensive, multi-level prevention strategies within school settings.

Although associations between bullying and adolescent risk behaviors have been documented internationally, evidence from Ecuador remains limited. This study contributes regionally relevant data by documenting the prevalence of school bullying and cyberbullying and their associations with mental health and behavioral outcomes among Ecuadorian adolescents. Within a context characterized by limited mental health services and developing school-based prevention programs, these findings offer valuable insights for policymakers and practitioners designing culturally appropriate interventions.

## 5 Conclusions

The findings highlight the urgent need for targeted interventions addressing bullying and its associated risks, particularly suicidal behaviors and alcohol use. Prevention efforts should extend beyond individual-level approaches and involve students, families, teachers, and school systems. Given its substantial impact on adolescent mental health, bullying should be treated as a serious public health issue requiring proactive, coordinated responses to promote safe and supportive school environments.

## 6 Limitations

This study has several important limitations. First, the cross-sectional design precludes causal inference or temporality between peer victimization and outcomes. While multivariable analyses adjusted for age and sex confirmed robust associations, residual confounding by unmeasured factors—such as socioeconomic status, family environment, prior mental health history, academic performance, or co-occurring adversities—remains a major concern, as these were unavailable in the YRBSS dataset.

Second, bullying and cyberbullying were assessed using single-item, dichotomous (yes/no) measures, standard in YRBSS surveillance but limited in construct validity. These binary indicators fail to capture victimization frequency, severity, duration, or modality (physical, verbal, relational, cyber-specific), potentially diluting effect estimates and masking dose-response patterns. Psychometrically validated multi-item scales are needed in future research.

Third, self-reported data risks recall bias, social desirability (especially for suicide/substance use), and misclassification. The convenience school sample limits generalizability, excluding dropouts or home-schooled youth potentially at higher risk.

Despite these limitations, this study provides valuable preliminary evidence on peer victimization correlates using standardized measures in an under-researched Ecuadorian context, justifying more robust longitudinal research.

## Data Availability

The raw data supporting the conclusions of this article will be made available by the authors, without undue reservation.
